# An Analysis of Open-Ended Coaxial Probe Sensitivity to Heterogeneous Media

**DOI:** 10.3390/s20185372

**Published:** 2020-09-19

**Authors:** Christopher L. Brace, Sevde Etoz

**Affiliations:** 1 Departments of Radiology and Biomedical Engineering, University of Wisconsin, Madison, WI 53705, USA; 2 Department of Cardiology, Johns Hopkins University, Baltimore, MD 21218, USA; setozni1@jhmi.edu

**Keywords:** dielectric property measurement, open-ended coaxial probe, biological tissues, heterogeneous media

## Abstract

Open-ended coaxial probe spectroscopy is commonly used to determine the dielectric permittivity of biological tissues. However, heterogeneities in the probe sensing region can limit measurement precision and reproducibility. This study presents an analysis of the coaxial probe sensing region to elucidate the effects of heterogeneities on measured permittivity. Coaxial probe spectroscopy at 0.5–20 GHz was numerically simulated while a homogenous background was perturbed with a small inclusion of contrasting permittivity. Shifts in the measured effective permittivity provided a three-dimensional assessment of the probe sensitivity field. Sensitivity was well-approximated by the square of the electric field for each analyzed probe. Smaller probes were more sensitive to heterogeneities throughout their sensing region, but were less sensitive to spectral effects compared to larger probes. The probe sensing diameter was less than 0.5 mm in all directions by multiple metrics. Therefore, small heterogeneities may substantially impact permittivity measurement in biological tissues if located near the probe-tissue interface.

## 1. Introduction

Accurate measurement of the microwave-range dielectric permittivity in biological materials is critical to many fields, including therapeutic energy delivery, imaging, patient monitoring and safety dosimetry. Among the techniques for measuring permittivity, reflectometry with an open-ended coaxial probe has been a mainstay due to its broadband operation and ability to measure biological tissues without manipulation or destruction [1]. Open-ended coaxial probes can also be used for thermally stable measurements at over 100 °C [2,3,4].

The basic process for permittivity measurement is to collect complex reflection coefficients ($S_{11}$) from the probe-medium interface over the frequency of interest using a network analyzer. Probe calibration is performed by using three standards with known reflectance (often open, short and deionized water) before $S_{11}$ data from a test sample are collected. The sets of $S_{11}$ data are then converted to complex permittivity using an analytic or numerical model of the coaxial probe [5]. Coaxial probes for biological tissue measurement are typically 1–10 mm in diameter, though smaller probes have been described [6].

Coaxial probe models assume that the sample medium is homogenous and isotropic within a probe sensing volume. Early data compiled into the well-cited series of papers by Gabriel et al. were collected under this assumption [7,8,9]. However, sensing volume is not an objectively defined concept; the impact of small variations in material permittivity has received less attention in the literature overall. Hagl et al. determined that the minimum homogenous sample size to avoid errors from boundary reflections should be 3–5 mm axially by 5–11 mm radially, depending on probe size and the permittivity of the sample medium [10]. Later, Meaney et al. used an experimental setup with layered media to suggest that the measured permittivity is disproportionately affected by media within 200–400 μm (i.e., the measurement volume is less than 1 mm) [11]. La Gioia et al. used a series of simulations and measurements in tissue to demonstrate that the spatial distribution of materials around the probe alters the measured permittivity, and determined that the measurement volume was at most 1 mm [12]. While there is some qualitative agreement between these studies, a quantitative functional relationship between heterogeneous material permittivity and the effective permittivity measured by a coaxial probe has not yet been established.

The objective of this study was to quantitatively map the sensitivity of an open-ended coaxial probe to nearby variations in permittivity using numerical simulations. We hypothesized that the effective permittivity measured by the probe is a weighted average of the permittivity in the sampling region, and proposed that the weighting function is related to the intensity of the electric field emitted by the probe. The weighting function may be interpreted as the probe sensitivity field to facilitate a more complete analysis of probe measurement volume.

## 2. Materials and Methods

### 2.1. Numerical Model Setup

The basic technique for determining a probe sensitivity field was to numerically simulate permittivity measurements in a homogenous background perturbed with a small dielectric inclusion. Simulations were performed in commercial software implementing the finite-element method (COMSOL Multiphysics^®^ v5.3a, Stockholm, Sweden). The numerical procedure reproduced the experimental procedure described in the introduction. In brief, reflection coefficients from the input port ($S_{11}$) were determined from 0.5 to 20 GHz in 0.25 GHz intervals in calibration and test media. Complex permittivity was computed from $S_{11}$ data by using a rational function model of the coaxial probe admittance [13].

**Figure 1:**
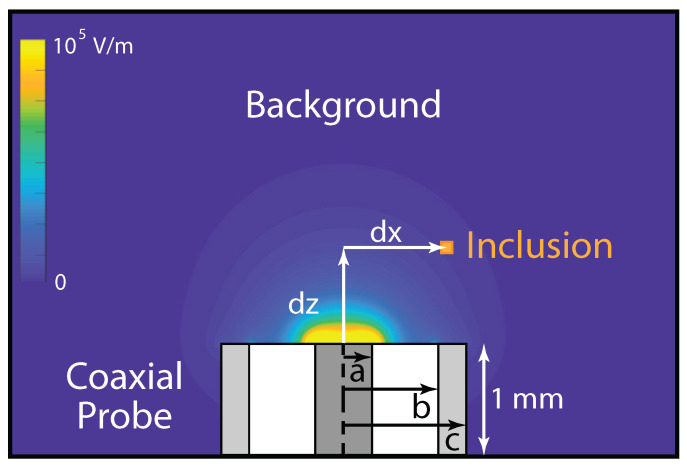
Experimental setup and parameters. A small, cubic inclusion with permittivity contrasting to the background was swept through *x*, *y*, and *z* directions around an open-ended coaxial probe. Background colormap illustrates the probe electric field magnitude assuming a 1 W input power. Color scale is linear.

The geometric model comprised a three-dimensional open-ended coaxial probe (Figure 1). Four probe diameters were analyzed—1.3, 2.2, 3.6 and 6.4 mm—corresponding to commonly available 50 Ω semi-rigid coaxial cable (Table 1). The coaxial center and outer conductors were assumed to have perfectly electrically conducting surfaces. The coaxial dielectric was polytetrafluoroethylene (PTFE; $\epsilon_{r}$ = 2.05, σ = $10^{-6}$ S/m). The probe was assumed to be immersed 1 mm into the background with a coaxial input port on the dielectric face. $S_{11}$ analyses were conducted from the probe-material interface.

The background domain was a cylinder with a 12 mm diameter and 10 mm height with scattering external boundaries. A probe without a flange is should normally be immersed at least 5 mm into the test material; however, because the exterior boundaries were minimally reflecting, the domain appeared as near-infinite to the port, so that a shorter probe could be utilized. The inclusion was assumed to be a cube with varying side lengths as described in Section 2.4. Preliminary analyses verified that the selected probe length, domain size, and boundary conditions reduced computational time compared to a larger domain without altering permittivity measurement accuracy by more than ±0.5%.

**Figure 2:**
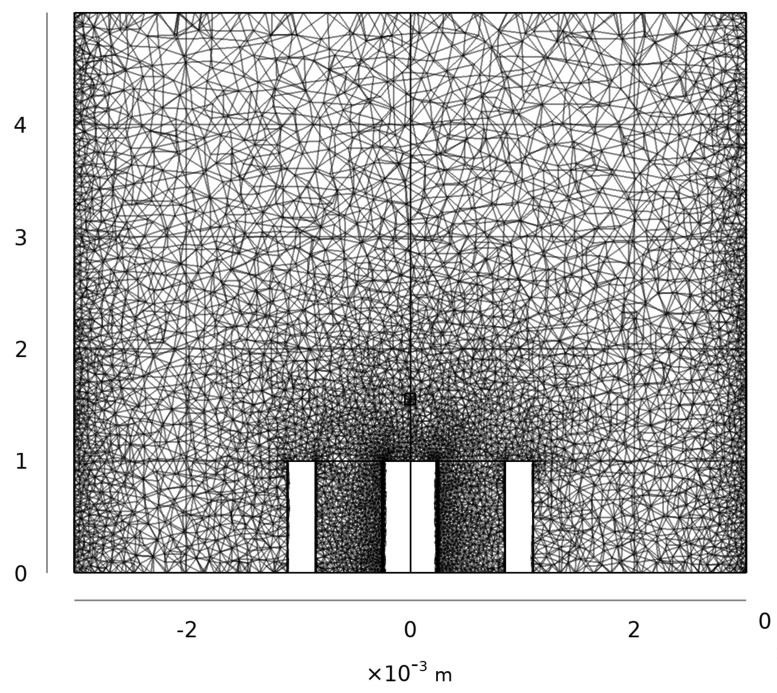
Cross-sectional view of the finite element mesh for an inclusion 0.1 mm on a side. Unmeshed areas represent the metallic center conductor and outer conductor domains.

Domains were filled with tetrahedral mesh elements with a maximum size of 0.2 mm to accommodate simulations up to 20 GHz in the assumed permittivity. Adaptive mesh refinement was utilized to provide finer meshing around sharp features that created high field gradients. Each geometry contained around 3–5 $\times 10^{5}$ elements (Figure 2). Mean computation time for each geometric variation was 748 s on a 16-core 2.5 GHz Xeon CPU with 128 GB available memory. Parametric simulations were distributed across 160 nodes of a SLURM-managed cluster to reduce overall computational time.

### 2.2. Calibration Verification

Open, short and water calibration standards were simulated by assigning the permittivity of the background and inclusion domains as vacuum, perfect electrical conductor (PEC), or deionized water [14]. While a single set of $S_{11}$ data from each calibration standard could be used to more rapidly evaluate all test conditions, changes in inclusion location or size altered the finite-element mesh for a given probe, which led to numerical variation in the calculated $S_{11}$. A preliminary analysis was conducted to determine the error introduced with a single calibration set compared to performing specific calibrations for each test condition.

The permittivity of a test medium comprising the background and a 0.5 mm inclusion (no contrast) was determined from the simulated $S_{11}$ of a 2.2 mm diameter probe from 0.5–20 GHz. The inclusion was centered at permutations of x=0,0.2,0.5 or 1 mm and z=0,0.2,0.5 or 1 mm. For each inclusion location, two different calibration sets were evaluated: (a) a single set of open, short and water calibration $S_{11}$ from x=z=0 or (b) a set of calibration $S_{11}$ for the specific inclusion location. The permittivities calculated using each calibration approach were compared to the known permittivity of the background and inclusion.

The single set of calibration data created errors up to 5.7% in $\epsilon_{r}$ calculations, and 5.9% in σ calculations, across frequencies. Errors were reduced to less than 0.9% and 1.1%, respectively, when using calibration data specific to the test condition. Therefore, calibrations specific to the inclusion location and size were used for all test conditions despite the increased computational time. These results can also be interpreted as indicating that the numerical error in the permittivity calculation was 1%.

### 2.3. Perturbation Analysis

The primary objective of this study was to spatially map the sensitivity of each coaxial probe to perturbations in the permittivity of the adjacent medium. The working hypothesis was that the effective permittivity measured by the coaxial probe, $\epsilon_{\mathrm{eff}}$, would be an average of the spatially varying permittivity of the test medium, ϵ(x,y,z), weighted by a function w(x,y,z), that corresponds to the sensitivity field of the probe:

(1) $$\epsilon_{\mathrm{eff}}=\frac{\underset{R}{\;\int \int \int \;}\epsilon \left(x,y,z\right)w\left(x,y,z\right)dV}{\underset{R}{\;\int \int \int \;}w\left(x,y,z\right)dV},$$

where *R* defines a region around the probe of which bounds should extend at least to the extents of the expected measurement volume.

To determine w(x,y,z), we perturbed a background of higher permittivity ($\epsilon_{\mathrm{high}}$) with a single dielectric inclusion of lower permittivity ($\epsilon_{\mathrm{low}}$) and observed the impact on measured effective permittivity ($\epsilon_{\mathrm{eff}}$). The inclusion was swept through the background in 10–500 μm steps, with smaller step sizes near corners in the probe to help resolve high sensitivity gradients. Each test condition included analysis of six domain material combinations: three calibration standards as described in Section 2.2, a homogenous domain of high permittivity, a homogenous domain of the low permittivity, and the test condition of a high-permittivity background with a low-permittivity inclusion. The frequency-dependent complex relative permittivity was determined using a Cole–Cole relaxation model [9]:

(2) $$\epsilon_{r}=\epsilon_{\infty }+\frac{\epsilon_{s}-\epsilon_{\infty }}{{\left(1+i\omega \tau \right)}^{1-\alpha }}+\frac{\sigma_{s}}{j\omega \epsilon_{0}}$$

where $\epsilon_{\infty }$ is relative permittivity at high frequencies, $\epsilon_{s}$ is static relative permittivity, ω is angular frequency (rad/s), τ is the relaxation rate (1/s), α is a dispersion parameter (0<α<1), and $\sigma_{s}$ is the static ionic conductivity (S/m). The higher permittivity was assumed to be similar to normal saline (Table 2). The lower permittivity was created by scaling the higher permittivity by a contrast factor, ξ.

The high-permittivity and low-permittivity background media served as reference values to calculate the inclusion influence, *I*, on the effective permittivity measured from the test medium, $\epsilon_{\mathrm{test}}$,

(3) $$I\left(x,y,z\right)=\left|\frac{\epsilon_{\mathrm{high}}-\epsilon_{\mathrm{test}}}{\epsilon_{\mathrm{high}}-\epsilon_{\mathrm{low}}}\right|,$$

When inclusion size is small enough to adequately resolve the sensing region (less than 1/2 the center conductor radius), *I* can be interpreted as a spatial mapping of coaxial probe sensitivity to dielectric heterogeneities (i.e., a probe sensitivity field). Here *I* is formulated from the magnitude of a complex-valued ratio; it could also be formulated for the relative permittivity and conductivity separately.

### 2.4. Inclusion Size Analysis

In a preliminary study, we sought to determine the effect of inclusion size on influence mapping resolution. Inclusions of 0.01 to 1.0 mm were evaluated on a grid of *x* and *z* positions around the coaxial probe with ξ = 0.5. Inclusion influence was strongly and negatively proportional to inclusion size, as noted in a preceding study [15]. While larger inclusions created more substantial changes in measured permittivity, they produced very low-resolution maps of the influence relative to inclusion location. The smallest inclusion side length, 0.01 mm, still generated measurable perturbations in probe $S_{11}$ so was used for spatial analysis of influence throughout the remainder of the study.

### 2.5. Sensing Region Metrics

The expected spatial variation in influence suggests that a probe’s sensing region cannot be fully characterized by a single metric (e.g., volume or diameter). However, recognizing that such metrics have practical utility in comparing devices or setting device measurement expectations, we propose some that may be meaningful:

1. Normalized influence across probes, ${\tilde{I}}_{n}$, as influence divided by the maximum influence in the entire dataset:
  (4) $${\tilde{I}}_{n}=\frac{I_{n}}{\max(||I_{n}||\in \left\{n=1\ldots N\right\})},$$
  where *n* is the probe number and *N* is the total number of probes. This normalization removes the effects of inclusion size or geometry, inclusion-background contrast, background permittivity, or probe input power on influence to facilitate comparison of sensitivity across probes and frequencies.
2. Normalized influence within a probe, ${\hat{I}}_{n}$, as influence divided by the maximum influence for a given probe:
  (5) $${\hat{I}}_{n}=\frac{I_{n}}{\left|\right|I_{n}\left|\right|},$$
  where *n* is the probe number. This normalization is useful for comparison of influence to probe-specific features such as the emitted electric field.
3. Cumulative influence, $I_{\mathrm{cum}}$, as the integration of influence over the sensing region, *R*:
  (6) $$I_{\mathrm{cum}}=\frac{1}{V}\int \;\;\;\int_{R}\;\;\;\int I\left(x,y,z\right)dV.$$
  $I_{\mathrm{cum}}$ describes a probe’s overall sensitivity to material heterogeneities but does not include spatial information.
4. ${\tilde{I}}_{n}$ contour dimensions in the axial (x=0) and radial (z=0) directions, $D_{\mathrm{ax}}$ and $D_{\mathrm{rad}}$, respectively. The selection of contour level does not have precedent in the literature so we selected 1% for this analysis (i.e., the contour where influence changes by 1% relative to $max(||I_{n}||\in \left\{n=1\ldots N\right\})$.
5. ${\tilde{I}}_{n}$ isosurface volume based on the same 1% contour.

## 3. Results

### 3.1. Inclusion Influence Metrics

Changes in calculated permittivity and conductivity were on the order of $10^{-5}$ for a 0.01 mm inclusion and were detectable when using the geometry-specific calibration procedure. Influence maps demonstrated substantial variation with respect to inclusion location, measurement frequency, and probe geometry; only slight variation was noted with respect to background permittivity. Dielectric contrast between the background and inclusion was not a significant factor in the analysis. More detailed analysis of these parameters follows.

**Figure 3:**
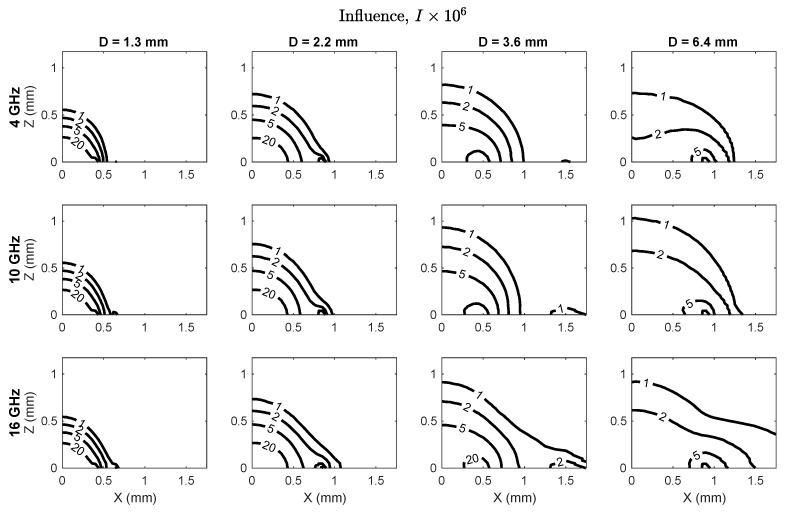
Influence in the xz-plane, where x=0 coincides with the probe symmetry axis and z=0 lies along the probe measurement plane. Influence peaked near the outer edge of the center conductor at z=0 for all probes.

Influence maps are shown in Figure 3 for three frequencies (4, 10 and 16 GHz) to illustrate trends over the 0.5–20 GHz spectrum that was evaluated. The maximum influence for each probe was observed at the interface of the center conductor, coaxial dielectric and adjacent material, and was inversely related to probe diameter (Table 3). Influence decayed nonlinearly from the peak location for each probe, with the rate of decay dependent on probe size and frequency. The size of the zone of highest influence and the influence gradient were both inversely related to probe size.

**Figure 4:**
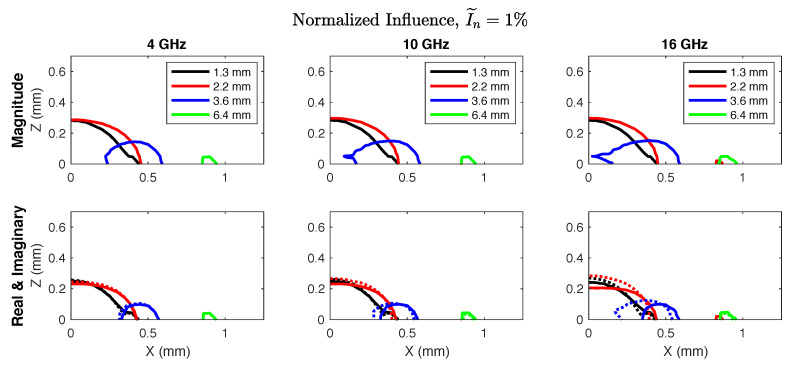
Normalized influence in the xz-plane, where x=0 coincides with the probe symmetry axis and z=0 lies along the probe measurement plane. The top row shows influence magnitude, while the bottom row shows influence in the real (solid lines) and imaginary (dotted lines) parts. The measurement volume defined by the 1% contour was inversely related to probe size and centered around the outer radius of the coaxial center conductor. There was some spatial separation in the influence on real and imaginary parts at frequencies above 10 GHz.

Quantitative sensing region metrics are compiled in Table 3. Overall, there was only slight variability in the metrics from 0.5–20 GHz so the data are presented as an average ± standard deviation across frequencies. We note that the tabulated metrics were drawn from influence calculated by Equation (3), but similar values were observed for influence on the real and imaginary parts representing relative permittivity, $I_{\epsilon }$, and conductivity, $I_{\sigma }$, individually (Figure 4).

### 3.2. Sensitivity Field Analysis

Influence demonstrated a spatial variation qualitatively resembling the expected electric field magnitude around an open-ended coaxial probe. Under the hypothesis that the influence would be related to the electric field, we sought to determine a best-fit between influence and electric field. Both variables were normalized to their respective maxima for each probe and frequency (${\hat{E}}_{n}=|\vec{E}{|}_{n}/\max(|\vec{E}{|}_{n})$). The relationship between influence and electric field intensity was assumed to be described by a power law:

(7) $$F_{n}=q_{n}{\hat{E}}_{n}^{\alpha_{n}}.$$

**Figure 5:**
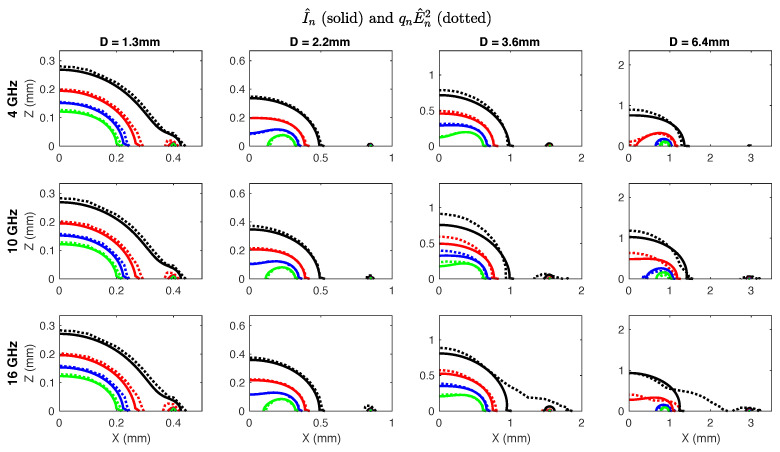
Overlay of ${\hat{I}}_{n}$ (dotted lines) and $q_{n}{\hat{E}}^{2}$ (solid lines) at levels of 0.5 (black), 1 (red), 2 (blue) and 3.5 (green) to illustrate curve fitting. Overall fit was very good at lower frequencies and with smaller probes. More observable discrepancies in fit can be seen with 3.6 and 6.4 mm probes at frequencies over 10 GHz.

Therefore, the objective was to determine the parameters $q_{n}$ and $\alpha_{n}$ that minimized mean absolute error (MAE) between ${\hat{I}}_{n}$ and $F_{n}$ in a least squares sense. Optimal fitting parameters for each probe diameter and selected frequencies are provided in Table 4. For most cases $\alpha_{n}\approx 2$ so a second optimization was conducted with a fixed $\alpha_{n}=2$. The optimal values of *q* averaged over frequency are provided in Table 4. While MAE was slightly higher overall for $\alpha_{n}=2$, the fit between influence and electric field squared was very good (Figure 5).

Since ${\hat{I}}_{n}\approx q_{n}{\hat{E}}^{2}$, the weighting function w(x,y,z) of Equation (1) can be reasonably approximated by the square of the electric field emitted by a given probe. This implies that the integration region, *R*, can be practically bounded a few millimeters from the probe without expecting negative effects from boundary reflections since the electric field decays to a negligible level within around 1 mm of the probe center (Figure 5).

### 3.3. Inclusion Contrast and Background Effects

**Figure 6:**
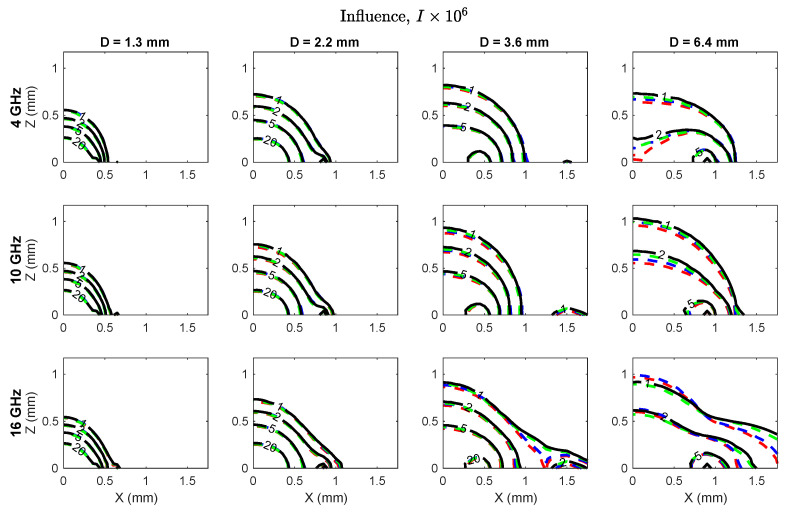
Influence maps for four combinations of background coefficient and contrast levels: 1.0 and 0.5 (solid black), 1.0 and 0.75 (dotted green), 0.75 and 0.5 (dotted blue), 0.75 and 0.75 (dotted red). Contrast had minimal impact on influence, while changes due to background permittivity were commensurate with changes in the electric field intensity around the probe.

Background permittivity and inclusion contrast had little impact on inclusion influence, particularly for the 1.3 and 2.2 mm probes and at frequencies less than 10 GHz. For the 3.6 and 6.4 mm probes at frequencies over 10 GHz the change in influence due to background permittivity was mirrored by changes in electric field intensity (Figure 6).

### 3.4. Validation Testing

The validity of the weighted-average model in Equation (1) was tested using the same numerical simulation procedure. The inclusion was removed and the background permittivity was defined using either a homogenous or heterogeneous distribution. The heterogeneous distribution was created by defining a Gaussian random field coefficient, GRF(x,y,z) (see Appendix A), to scale the static permittivity from Equation (2):

(8) $$\epsilon_{s}\left(x,y,z\right)=0.95+0.05GRF\left(x,y,z\right)\epsilon_{s,\mathrm{baseline}}.$$

The resulting permittivity varied spatially by ±20% relative to the assumed baseline permittivity value [16]. Homogenous permittivity fields of $\epsilon_{s}=30-50$ at intervals of five, and twenty randomized fields with $\epsilon_{s,\mathrm{base}}=45$ were generated. The effective permittivity of each sample was measured as described in Section 2.1. The numerically measured values were compared to (1) the unweighted average of the permittivity field and (2) the weighted average based on the probe electric field outlined in Section 3.2. The weighting function parameters were also optimized to minimize errors between weighting function estimates and numerical measurements.

**Figure 7:**
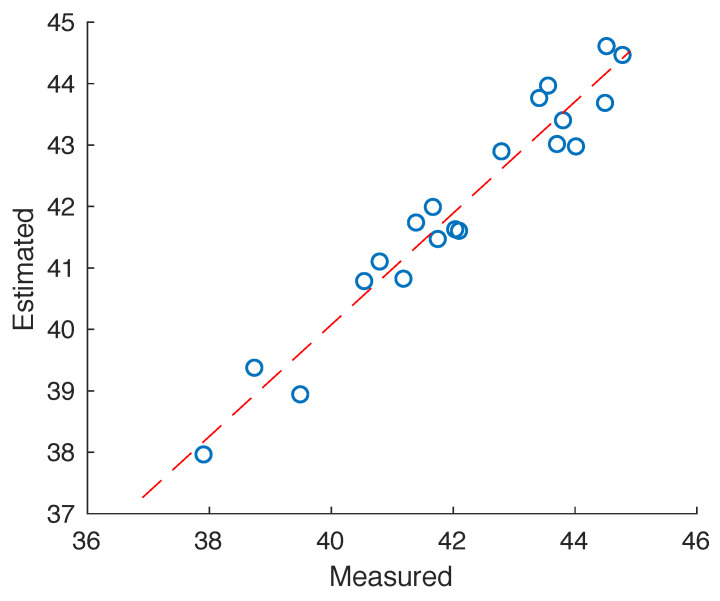
Permittivity estimated from the ${\hat{E}}_{n}^{2}$ weighted average compared to those measured from numerical simulations. A strong positive correlation was confirmed using Spearman’s method (p< 0.001, $R^{2}$ = 0.98).

Estimates based on the weighted-average model had significantly less error compared to numerical measurements than the unweighted volume average of the permittivity field (Table 5). The optimal weighting function exponent was α=3.3, though α=2 provided an acceptable agreement (Figure 7). The improved fit for higher exponents suggests even greater influence of permittivity variations immediately adjacent to the probe.

## 4. Discussion

Characterization of biological tissue permittivity has been hampered by a poor understanding of how tissue heterogeneities impact open-ended coaxial probe measurements. In particular, the sensitivity field of the coaxial probe has not been adequately determined, leading to uncertainty about the size of the probe sensing region as well as the influence of small heterogeneities on permittivity. This study addressed both sources of uncertainty. Probe sensitivity fields were determined for four common coaxial cable sizes by using numerical simulations in the presence of a perturbing dielectric inclusion. The sensitivity field decayed rapidly away from the center conductor and was roughly proportional to a power function of the electric field emitted by the coaxial probe. Simulations demonstrated that the measured effective permittivity can be predicted from a weighted average of the heterogeneous permittivity.

The observed trends were relatively consistent across frequencies and probe sizes, with some exceptions. The greatest influence was seen in the medium adjacent to the outer radius of the center conductor, so probe size was a factor in the pattern and magnitude of sensitivity. This appeared consistent with the findings of Sarolic and Matkovic, who observed that a larger probe diameter was associated with deeper electric field penetration into a layered medium but that the surface layer was still the dominant influence on measured permittivity [17]. There were also some slight differences in sensitivity to the real and imaginary components of permittivity among larger probes and higher frequencies (Figure 4). This implies that even small heterogeneities near the axis of the coaxial center conductor may introduce variations in measured tissue conductivity at frequencies above 10 GHz. It is possible that tissue heterogeneity could be quantified by observing such high-frequency variations.

There is not a common definition of sensing volume so metrics for assessing probe sensing region were proposed. In the context of biological material measurement, Meaney et al. estimated that the sensing volume of a similar open-ended coaxial probe was ∼0.3–0.4 mm based on measurements of layered media [18]. La Gioia et al. determined the sensing volume was less than 1 mm in one report, and was 0.5 mm for a dielectric contrast of 0.5 in another report [12,19]. While different in approach and definition, our observation that the inclusion influence of each probe decayed to less than 5% of its maximal value by around 0.4 mm from the center conductor parallels the estimates from those prior experimental and theoretical works. In contrast to LaGioia et al., we did not find that the probe sensitivity changed substantially with a contrast factor [19]. Negligible influence was noted at distances greater than 3 mm from the probe center, which is also concordant with the findings of Hagl et al. that samples should be at least that size to avoid boundary effects corrupting the measurement [10].

The existing literature on tissue properties measured with open-ended coaxial probes is substantial. As noted in prior reviews, reported results include measures of variance or repeatability, but the sources of variability have not always been appreciated since the sensitivity of the measurement devices to dielectric heterogeneity has not been well-understood [5]. A prior study of the open-ended coaxial probe showed that even sub-millimeter inclusions can generate measurable changes in effective permittivity [15]. The present study extends our understanding of how even micron-scale heterogeneities can influence the measured permittivity. In particular, the spatial relationship of the heterogeneities to the probe is an important aspect that is not captured by, for example, treating the material as a uniformly heterogeneous mixture [20]. In future studies, it may be important to use imaging or other detection techniques to assess tissue heterogeneities concurrently with permittivity measurement [15]. Alternative probe designs that are less-sensitive to heterogeneities may also be more appropriate for some measurements [1,21,22].

There were inherent limitations in this study. The numerical technique allows for controlled and highly sensitive measurement of small inclusions but relies on idealized probe geometries and tissue parameters. Probe manufacturing tolerances are a source of experimental error that was not considered with our approach [23]. Additional work is necessary to experimentally validate these findings. In addition, the parameter space was limited. The low permittivity media was constructed by multiplying the high permittivity by a contrast factor, ξ. Therefore, both the real and imaginary parts were scaled by the same degree. Additional analysis would be required to determine if all of the same conclusions can be drawn for a wider range of material properties. Other probe designs, frequency spectra, and even permittivity calculation techniques could require additional consideration. The dielectric perturbation technique introduced in this study could be applied in future numerical analyses of other parameters. The numerical technique may also be a template for experimental methods in future studies. However, because changes in measured permittivity induced by the inclusion were well under the measurement sensitivity of current systems, experimentally reproducing the analysis technique may not be practical at this scale. Finally, the comparison of measured permittivity in heterogeneous media was not comprehensive. Changing the “measurement volume” over which the volume averages were obtained could change the accuracy of their estimates. What we demonstrated was simply that inclusion influence (i.e., probe sensitivity) was related to the square of the radiated electric field that, in turn, proved useful to construct a weighted-average estimate of the permittivity measured from a heterogeneous media.

## 5. Conclusions

In conclusion, this study introduced a dielectric perturbation technique to assess coaxial probe sensitivity during permittivity measurements. Probe sensitivities were found to be well-described by a power function of their normalized electric fields. The measured effective permittivity could be reasonably predicted as a weighted average of the adjacent permittivity field by using the electric field power function for weighting.

## Figures and Tables

**Table 1:** Coaxial probe dimensions, expressed in millimeters. Variables *a*, *b* and *c* represent the radii of the center conductor, coaxial dielectric and outer conductor, respectively (Figure 1).

Coaxial Diameter	a	b	c
1.3	0.12	0.40	0.65
2.2	0.26	0.85	1.1
3.6	0.47	1.55	1.8
6.4	0.89	2.95	3.2

**Table 2:** Material parameters used to simulate the higher permittivity background material, similar to normal saline.

Material	$\epsilon_{\infty }$	$\epsilon_{s}$	τ (ps)	α	$\sigma_{s}$ (S/m)
Background	78.6	4.2	9.31	0.013	0.2

**Table 3:** Measurement volume metrics by coaxial probe diameter. Values expressed as mean ± standard deviation over 0.5–20 GHz. Diameters expressed in micrometers. Volume expressed in cubic millimeters. Normalized metrics are dimensionless.

Probe	Max |I|	$I_{\mathrm{cum}}\times 10^{3}$	$D_{\mathrm{ax}}=1\%$	$D_{\mathrm{rad}}=1\%$	V=1%
1.3 mm	$1.6\times 10^{-3}$	3.33 ± 0.03	450 ± 0	577 ± 8	0.083 ± 0.00
2.2 mm	$1.0\times 10^{-3}$	3.77 ± 0.13	479 ± 30	591 ± 16	0.130 ± 0.01
3.6 mm	$1.1\times 10^{-4}$	2.84 ± 0.25	457 ± 95	305 ± 10	0.121 ± 0.01
6.4 mm	$7.4\times 10^{-5}$	2.25 ± 0.35	103 ± 20	103 ± 8	0.022 ± 0.01

**Table 4:** Optimized power function parameters from Equation (7), expressed as $q_{n}/\alpha_{n}$ for each frequency with frequency-averaged coefficient ($\bar{q_{n}}$) for $\alpha_{n}=2$.

Probe	4 GHz	10 GHz	16 GHz	$\bar{q_{n}}$
1.3 mm	7.431/1.610	7.392/1.598	6.772/1.563	19.56
2.2 mm	2.869/1.890	3.799/1.842	3.815/1.840	4.916
3.6 mm	12.13/1.468	9.391/1.377	7.878/1.349	81.96
6.4 mm	1.493/1.671	1.722/1.598	1.031/1.584	3.494

**Table 5:** Mean percentage errors between permittivities measured in simulations and predictions from either volume averaging or ${\hat{E}}^{2}$-weighted averaging of the permittivity distribution.

Probe	Volume Average	Weighted Average
1.3 mm	4.25%	0.454%
2.2 mm	4.25%	0.890%
3.6 mm	4.25%	1.42%
6.4 mm	4.25%	2.06%

## Appendix A. Gaussian Randomized Field Generation

Randomized material parameters in three dimensions can be developed from a series of elementary waves of the form

$$A\cos(\vec{k}\cdot \vec{r}+\phi ),$$

where $\vec{k}$ is the wave vector, $\vec{r}$ is the position vector, and ϕ is the phase angle. To generate a random three-dimensional field, the phase angle can be randomly assigned from a uniform distribution on [−π/2,π/2]. The wave vector may be expressed in terms of spatial frequency components in cartesian coordinates as $\vec{k}=\left(k_{x},k_{y},k_{z}\right)=2\pi \left(\nu_{x},\nu_{y},\nu_{z}\right)$. For simulation purposes it is convenient to discretize the spatial frequencies as $\nu_{x}=m$, $\nu_{y}=n$, and $\nu_{z}=p$ with bounds ±(M,N,P) that balance spectral resolution and computational demand. |M,N,P|∈(10,20) was suitable for this study.

The amplitude of each wave component, $A_{mnp}$, was also randomly selected. There is not an established amplitude distribution for biological tissues so we assumed a power spectra of the form

$$A_{mnp}=\frac{1}{{\sqrt{m^{2}+n^{2}+p^{2}}}^{\beta }},$$

where β is a spectral exponent that defines the attenuation of higher frequencies. For reference, β=1 defines white noise while β=2 is Brownian noise. The final distribution of waves to create the randomized field can be expressed as

$$GRF\left(x,y,z\right)=\sum_{p=-P}^{P}\sum_{n=-N}^{N}\sum_{m=-M}^{M}A_{mnp}\cos\left(2\pi \left(mx+ny+pz\right)+\phi \left(m,n,p\right)\right).$$

## References

[1] Stuchly M.A., Stuchly S.S. (1980). Coaxial Line Reflection Methods for Measuring Dielectric Properties of Biological Substances at Radio and Microwave Frequencies—A Review. IEEE Trans. Instrum. Meas..

[2] Lazebnik M., Converse M.C., Booske J.H., Hagness S.C. (2006). Ultrawideband temperature-dependent dielectric properties of animal liver tissue in the microwave frequency range. Phys. Med. Biol..

[3] Ji Z., Brace C.L. (2011). Expanded modeling of temperature-dependent dielectric properties for microwave thermal ablation. Phys. Med. Biol..

[4] Lopresto V., Pinto R., Lovisolo G.A., Cavagnaro M. (2012). Changes in the dielectric properties of ex vivo bovine liver during microwave thermal ablation at 2.45 GHz. Phys. Med. Biol..

[5] La Gioia A., Porter E., Merunka I., Shahzad A., Salahuddin S., Jones M., O’Halloran M. (2018). Open-Ended Coaxial Probe Technique for Dielectric Measurement of Biological Tissues: Challenges and Common Practices. Diagnostics.

[6] McLaughlin B.L., Robertson P.A. (2009). Submillimeter Coaxial Probes for Dielectric Spectroscopy of Liquids and Biological Materials. IEEE Trans. Microw. Theory Tech..

[7] Gabriel C., Gabriel S., Corthout E. (1996). The dielectric properties of biological tissues: I. Literature survey. Phys. Med. Biol..

[8] Gabriel S., Lau R.W., Gabriel C. (1996). The dielectric properties of biological tissues: II. Measurements in the frequency range 10 Hz to 20 GHz. Phys. Med. Biol..

[9] Gabriel S., Lau R.W., Gabriel C. (1996). The dielectric properties of biological tissues: III. Parametric models for the dielectric spectrum of tissues. Phys. Med. Biol..

[10] Hagl D.M., Popovic D., Hagness S.C., Booske J.H., Okoniewski M. (2003). Sensing volume of open-ended coaxial probes for dielectric characterization of breast tissue at microwave frequencies. IEEE Trans. Microw. Theory Tech..

[11] Meaney P.M., Gregory A.P., Epstein N.R., Paulsen K.D. (2014). Microwave open-ended coaxial dielectric probe: Interpretation of the sensing volume re-visited. BMC Med. Phys..

[12] La Gioia A., Salahuddin S., O’Halloran M., Porter E. (2018). Quantification of the Sensing Radius of a Coaxial Probe for Accurate Interpretation of Heterogeneous Tissue Dielectric Data. IEEE J. Electromagn. Microw. Med. Biol..

[13] Anderson J., Sibbald C., Stuchly S. (1994). Dielectric measurements using a rational function model. IEEE Trans. Microw. Theory Tech..

[14] Nyshadham A., Sibbald C., Stuchly S. (1992). Permittivity measurements using open-ended sensors and reference liquid calibration-an uncertainty analysis. IEEE Trans. Microw. Theory Tech..

[15] Etoz Niemeier S., Brace C.L. Tissue permittivity measurement with concurrent CT imaging: Analysis of heterogeneity effects. Proceedings of the 13th European Conference on Antennas and Propagation (EuCAP).

[16] O’Rourke A.P., Lazebnik M., Bertram J.M., Converse M.C., Hagness S.C., Webster J.G., Mahvi D.M. (2007). Dielectric properties of human normal, malignant and cirrhotic liver tissue: In vivo and ex vivo measurements from 0.5 to 20 GHz using a precision open-ended coaxial probe. Phys. Med. Biol..

[17] Šarolić A., Matković A. Effect of the Coaxial Dielectric Probe Diameter on Its Permittivity Sensing Depth at 2 GHz–Simulation Study. Proceedings of the 2019 23rd International Conference on Applied Electromagnetics and Communications (ICECOM).

[18] Meaney P.M., Gregory A.P., Seppälä J., Lahtinen T. (2016). Open-Ended Coaxial Dielectric Probe Effective Penetration Depth Determination. IEEE Trans. Microw. Theory Tech..

[19] La Gioia A., O’Halloran M., Porter E. (2018). Modelling the Sensing Radius of a Coaxial Probe for Dielectric Characterisation of Biological Tissues. IEEE Access.

[20] Guihard V., Taillade F., Balayssac J.P., Steck B., Sanahuja J., Deby F. Modelling the behaviour of an open-ended coaxial probe to assess the permittivity of heterogeneous dielectrics solids. Proceedings of the 2017 Progress in Electromagnetics Research Symposium-Spring (PIERS).

[21] Meaney P., Rydholm T., Brisby H. (2018). A Transmission-Based Dielectric Property Probe for Clinical Applications. Sensors.

[22] Wang P., Brace C.L. (2012). Tissue Dielectric Measurement Using an Interstitial Dipole Antenna. IEEE Trans. Biomed. Eng..

[23] Popovic D., Okoniewski M. (2002). Effects of mechanical flaws in open-ended coaxial probes for dielectric spectroscopy. IEEE Microw. Wirel. Compon. Lett..

